# Secondary School Student Perceptions of Beginning Teachers’ Teaching Behaviours and Their Academic Engagement: Multilevel Modelling

**DOI:** 10.3390/bs16030399

**Published:** 2026-03-09

**Authors:** Ridwan Maulana, Michelle Helms-Lorenz, Cor Suhre

**Affiliations:** Department of Teacher Education, University of Groningen, Grote Kruisstraat 2/1, 9712 TS Groningen, The Netherlands

**Keywords:** teaching behaviour, student engagement, student perceptions, multilevel modelling

## Abstract

Past research has shown engagement during lessons to be pivotal for secondary education students to develop learning skills and to master curriculum objectives. Knowing which teaching behaviours matter most in creating sustainable student engagement is of the utmost importance for schools and school managers to be able to decide on the nature of the support beginning teachers need during their induction period to become competent teachers. Which dimensions of beginning teachers’ teaching behaviours are most in need of monitoring and support to guarantee active student engagement are still unclear. To provide some light on this issue, this study used data from a large database containing data of Dutch students’ perceptions of their teachers’ teaching behaviours during lessons, measured with the My Teacher Questionnaire, and data about self-reports of their own emotional and behavioural engagement during lessons. Our findings, based on multilevel analyses, indicate that differences between teachers’ classroom management skills and activating learning practices are the most salient components of teaching behaviour that impact the level of student engagement, regardless of student gender and family background. These findings suggest that, in general, students in Dutch secondary education seem to benefit in terms of academic engagement from efficient classroom management and more intensive and activating instruction practices of their beginning teachers.

## 1. Introduction

Today, class composition in secondary education has become more diverse than ever before (Maulana et al., 2023b). This requires teachers to be able to create a more responsive learning environment to address the learning needs of students and to ensure student engagement. Student engagement is a precondition for deep-level and meaningful learning (Skinner, 2016). However, a significant number of students gradually lose interest in schooling during their studies in secondary education and become disengaged (e.g., Cognia, 2022), which may not only be a detriment to their school achievements but can also lead to more serious consequences, such as school dropout and a false start to a professional career (Henry et al., 2012; Wang & Fredricks, 2014). To prevent this from happening, school teachers face the challenge of providing a motivating and productive learning environment that helps students to master school subjects that match their career interests and to develop skills to shape their future careers.

Until recently, most research on the improvement of student engagement has focused on the implementation of teaching practices to motivate students without focusing on the teaching behaviours that are essential in achieving successful implementation of these motivating practices (for a review, see Pedler et al., 2020). Little is known about which teaching skills need to be developed during teacher training to allow beginning teachers in secondary education to improve student engagement. Pre-service training for secondary education teachers typically focuses on basic teaching skills, such as providing a safe and stimulating learning climate, classroom management, and clarity of instruction, in the beginning of their professional practice, while other more complex teaching skills, such as activating learning, teaching learning strategies, and differentiating instruction, receive less focus and are often declared as areas for further skill development across their teaching careers (Maulana et al., 2015; van de Grift et al., 2014). To address this research gap, the present paper aims to provide insight into teaching skills that need more focus in teacher training by identifying the teaching skills of beginning teachers that best predict student engagement. We investigate this by assessing both students’ perceptions of beginning teachers’ teaching behaviours as well as the extent to which these teaching behaviours relate to students’ level of engagement in class. The purpose of this research is to improve teacher training and to ensure that beginning teachers will be more able to provide a responsive learning environment in order to stimulate student engagement.

Since supporting student learning depends highly on teachers’ teaching behaviours during lessons (Kyriakides et al., 2018; Maulana et al., 2017), understanding the relationships between teachers’ teaching behaviours and student engagement during lessons is important for stakeholders, particularly for school managers and teachers, to design and implement teaching practices that support students to become autonomous learners. It will also be useful for the design of tailored teacher education for prospective teachers and in-service learning arrangements for more experienced teachers. In this primarily explanatory study, we examine the relationship between student perceptions of teaching behaviour and their academic engagement during lessons in Dutch secondary schools. In doing so, we will also examine the differential effectiveness of essential dimensions of teaching behaviour for stimulating specific groups of students to actively engage in learning to achieve mastery of the learning objectives, as equity in the classroom setting is generally considered an important objective of school teaching.

## 2. Framework for the Current Study

### 2.1. Teachers’ Teaching Behaviours

The current study uses research on teaching and teacher effectiveness research, learning environment, and academic engagement as a theoretical framework for studying the link between teaching behaviour and student engagement. The framework recognises that the quality of teaching practices is a significant predictor of student learning and outcomes (Creemers & Kyriakides, 2008; Hattie, 2012; Maulana et al., 2023a).

Teaching behaviour is central in research on teaching quality. Based on reviews of evidence-based effective teacher behaviour research, van de Grift (2007) identified six observable dimensions of teaching behaviour. These dimensions include Safe and Stimulating Learning Climate, Efficient Classroom Management, Clarity of Instruction, Activating Learning, Differentiated Instruction, and Teaching Learning Strategies. The six dimensions of teaching behaviour are in line with other models of effective teaching behaviour and empirical findings (Danielson, 2013; Pianta & Hamre, 2009; Ko et al., 2013).

Safe and Stimulating Learning Climate (further called Learning Climate) refers to aspects of teacher–student relationships such as creating a safe and relaxing classroom climate for students to learn, showing respect to students and ensuring that students respect all classroom members, and encouraging self-confidence of students (Cornelius-White, 2007; Hattie & Clinton, 2008). Efficient Classroom Management (further called Classroom Management) refers to teaching behaviours related to ensuring that the lesson begins and ends on time, managing lesson transition efficiently, minimising time for task-unrelated matters, dealing with students’ misbehaviour efficiently, preparing the lesson well, and managing an efficient lesson structure (Marzano, 2003; Opdenakker & Minnaert, 2011; Yair, 2000). Clarity of Instruction covers aspects of instructional quality that are important for student learning, such as clear lesson structure, good interchange of explanations and lesson presentations, management of independent work, and clear assignment of individual and group works (Kindsvatter et al., 1988; Mortimore et al., 1988). Other behaviours include checking frequently that students understand the learning material well (Kame’enui & Carnine, 1998; Pearson & Fielding, 1991; Smith et al., 2008).

Activating Learning refers to behaviours that optimise student learning outcomes, such as applying active learning forms, intensifying instructions, and avoiding excessive work seats (Hampton & Reiser, 2004; Lang & Kersting, 2007). Other important aspects of Activating Learning include activating students’ prior knowledge, making use of “advance organisers,” and making sure that students recognise the relevance of the lesson content (Pressley et al., 1992; Nunes & Bryant, 1996). Differentiated Instruction refers to “a philosophy of teaching purporting that students learn best when their teachers effectively address variation in students’ readiness levels, interests, and learning profile preferences” (Tomlinson, 2005, p. 263). In order to apply Differentiated Instruction, teachers should recognise the diverse characteristics of their students they teach and understand that different students have different learning needs in order to progress in their learning. Teachers need to differentiate instruction by adapting and adjusting their teaching to the learning needs of their students. Teachers should emphasise the needs of each individual student when displaying instructional methods. The main goal of differentiated instruction is to maximise the learning potential of each student (Tomlinson, 2005). Some examples of teaching behaviours related to Differentiated Instruction include devoting extra time and additional instructions to weaker students, pre-teaching and re-teaching for students who need extra help, and implementing various teaching methods to ensure that all students are helped to achieve the desired outcomes (Houtveen et al., 1999; Kindsvatter et al., 1988; Lundberg & Linnakyla, 1993 Pearson & Fielding, 1991; Sijtstra, 1997).

Teaching Learning Strategies is “a heuristic that serves to support students, facilitating the development of internal procedures that enable them to perform the higher-level procedures” (van de Grift, 2007). The use of metacognitive strategies in teaching is an example of Teaching Learning Strategies. Metacognitive strategies provide a framework to help students achieve a higher level of learning skills (Carnine et al., 1988). One way of supporting the use of metacognitive strategies is scaffolding. Scaffolding is a form of temporary support provided by teachers (or by peers) that function as a bridge between students’ existing and desired skills.

These six dimensions of teaching behaviour include instructional behaviours that form the core of theoretical models of direct instruction and formative assessment, which are recognised as highly important for student learning and outcomes (Black & Wiliam, 2018; Maulana et al., 2015). The first three teaching behaviour dimensions, Learning Climate, Classroom Management, and Clarity of Instruction, constitute the basic teaching behaviours that should allow teachers to provide a solid teaching and learning environment for fostering student engagement in lessons. The teaching behaviours from the last three dimensions are central to providing a learner-centred education practice that could support the attainment of equity in the classroom. These teaching behaviours include using an inquiry style of teaching and the application of learning methods like cooperative learning to stimulate learners to effectively construct their understanding of school subject matter and to self-regulate their learning behaviours (Gillies, 2016). These teaching behaviours focus on academic growth support by providing instruction and opportunities for practice and feedback, as well as on student well-being by providing reassurance to struggling students that improvement is possible and by providing praise and encouragement for signs of learning and development. Especially the way teachers introduce learning tasks and apply formative assessment practices can foster students’ intrinsic motivation for engagement in deep learning to understand and master the content of a school subject, reduce evaluative pressure on students. and allow students a voice and choice in academic activities that suit student’s learning needs.

### 2.2. Student Perceptions of Teaching Behaviour

Studying teaching behaviour from the perspective of students contributes to the understanding of teachers’ teaching practices. Student perceptions are based on their (classroom) experiences over time, not merely from a single snapshot or limited number of observations. Student perceptions steer individual learning behaviours, based on their own judgements and insights. Research suggests that whether students will academically engage in and successfully complete the expected learning activities depends on their perceptions of the quality and integrity of teachers’ behaviours (den Brok et al., 2004). Student perception of teaching is a strong predictor of various learning outcomes (i.e., motivation, engagement, and achievement) (de Jong & Westerhof, 2001; Seidel & Shavelson, 2007). Compared to other methods, the use of questionnaires is considered more cost-effective and highly practical (Fraser, 1991) for measuring perceptions of teaching behaviours.

### 2.3. Student Engagement

Student engagement is a multidimensional construct. Skinner et al. (2009) made the conceptual distinguishment of two essential types of engagement for processing the lesson content: emotional and behavioural engagement. Fredricks et al. (2004) added cognitive engagement as a third component. Reschly and Christenson (2006) proposed a four-component model including academic, behavioural, cognitive, and psychological engagement. In the current study, we used the two-component model of Skinner et al. for several reasons. The emotional and behavioural components are foundational to understanding engagement (Appleton et al., 2008). Emotional and behavioural engagement are considered essential prerequisites for cognitive engagement (Hodges, 2020). Furthermore, cognitive and psychological engagement are regarded as less observable and include more indicators (Appleton et al., 2008). Including lengthy indicators is not preferred in survey research due to its risk of reducing response rates, a common phenomenon in many education systems, such as in the Netherlands.

Emotional engagement refers to students’ motivated emotions, such as enthusiasm, interest, and sense of identification with school (e.g., how students feel in the classroom and whether they enjoy learning new things, get involved, and show interest when they are working on something) (Fredricks et al., 2004; Wang & Holcombe, 2010). Emotional engagement is linked to willingness to learn and strengthening ties to school (school belongingness). Behavioural engagement refers to student actions and practices directed toward school and learning. The focus of behavioural engagement is on modes of conduct of academic, social, or extracurricular activities in terms of allocated mental resources and time spent on assignments. Research shows that both types of student engagement are important for their well-being in class and their achievements in school subjects (cf. Wong et al., 2024). Emotional and behavioural engagement are instrumental in achieving positive academic outcomes. Depending on a supportive learning environment, it can also help students in developing effective learning strategies and self-regulation, which are helpful for future learning (Li & Lerner, 2013).

In this study, we examine the link between both types of student engagement during lessons and teaching behaviours we consider essential for enhancing students’ autonomous learning development. Past research has shown a positive link between student engagement and student autonomous motivation (Maulana et al., 2016). Our framework for studying this link is based on self-determination theory (SDT), teaching and teacher effectiveness literature (e.g., Hattie, 2008; Muijs et al., 2014), and Black and Wiliam’s notions about formative assessment practices aiding students in becoming the owners of their own learning (Black & Wiliam, 2018).

### 2.4. Perceptions of Teaching Behaviours and Student Engagement in Lessons

SDT posits that students will generally be eager to engage in school learning when they perceive their teachers succeed in providing a classroom learning environment that meets their interests, focuses on their competence development, and allows for autonomy and relatedness in the classroom. Research by Kroeper et al. (2022) indicates that students’ readiness to engage in effective learning behaviours in class particularly depends on their perception of their teachers transferring growth mindset beliefs that give them confidence that they will be able to understand and master what is being taught. This perception needs to be grounded in students’ observations of effective teaching behaviours in a safe learning environment that prevents especially weaker students and more disadvantaged students from experiencing too much pressure to achieve the learning objectives at the same rate as stronger peers, as this might harm their egos and result in disengagement (e.g., den Brok et al., 2004). To promote deep understanding by all students in the classroom and to develop their readiness to engage in self-regulated learning to achieve mastery of the learning objectives, teachers need to adopt a responsive pedagogy. Core teaching behaviour entails eliciting student thinking during instruction and providing students with assignments to become aware of their proximity to obtaining the learning objectives. This enables teachers to decide on tailored suggestions to specific groups of students about effective learning strategies to improve their achievements (Black & Wiliam, 2018, Orr & Bieda, 2023). In general, the literature highlights the importance of teaching behaviours for student engagement, but it remains unclear which specific teaching behaviours matter more than other behaviours. The current paper tries to address this issue.

### 2.5. Student Background Factors Affecting Student Engagement

How students engage in learning during classroom settings not only depends on teachers’ behaviours in class but also on their initial readiness to process their teachers’ instructions and their ability to complete assignments. Research has shown that several student characteristics may affect student level of engagement directly or moderate the relationship between teacher behaviours and student engagement. Parental socioeconomic status, most commonly measured by parental level of education, is a powerful predictor of students’ attitudes towards schooling, school achievement, and dropout from upper secondary education (e.g., Lamb, 2011; Rumberger, 2011). The majority of the literature on the effects of parental level of education shows that these effects originate in different ways. Parental level of education not only influences the creation of educational and economic opportunities for their children but also parental involvement in education-supportive activities (Benner et al., 2016; Dubow et al., 2009; Kalil et al., 2012).

It is assumed that college-educated parents spend more time with their children, model achievement-oriented behaviour, provide opportunities for their children to engage in achievement-oriented experiences, engage in age-appropriate activities, and cultivate their children’s talents. Fan and Williams (2010) observed that parents who were involved in student learning, including providing advice regarding important learning decisions, communication with teachers, and keeping in contact with the school, predicted whether students spent time studying, worked hard, and persisted when facing difficulties. Although the nature of support does not differ substantially between students from parents of different levels of education, when children move from primary education to secondary education, the quality of support that parents can provide to their children often depends on the parental level of education, which may affect student readiness and motivation to engage in learning. Student engagement may also depend on student gender (Lam et al., 2012). Santos et al. (2021) reported higher student engagement among female students compared to male students and less engagement among older students. When students grow older, their academic engagement tends to deteriorate (Opdenakker et al., 2012).

### 2.6. School and Curriculum Factors Affecting Student Engagement

Given that curricula are largely content specific, and teacher guides for textbooks are differentially elaborated in different school types and school subjects (Remillard, 2005), differences in teaching behaviours and student engagement across school types and school subjects are expected.

**Figure 1:**
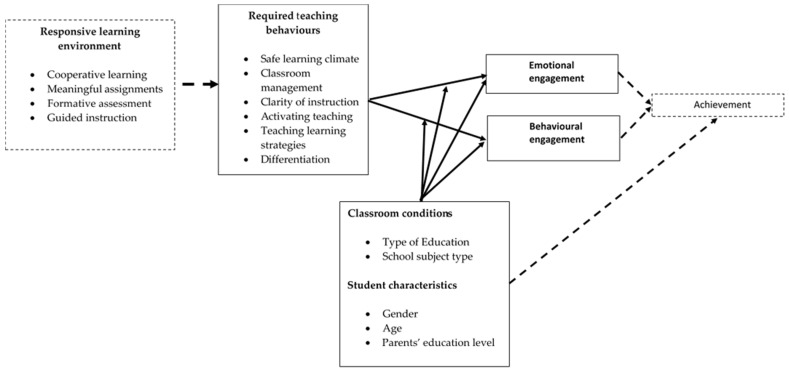
Conceptual model depicting the relationship between teaching behaviours, student engagement, learning outcomes, and background variables. (Solid line = tested variables, dashed line = not tested variables).

Figure 1 summarises a conceptual model of how our study views the impact of teachers‘ teaching behaviours during lessons on student engagement in class and how it would contribute to their achievements in school subjects, taking into account student and contextual characteristics.

The dashed boxes and arrows represent relevant concepts to teaching behaviours and student engagement, but they were not measured and tested in the current study.

## 3. The Current Study

The present study reports findings about the link between student perceptions of teaching behaviours and their engagement in lessons. The study context is secondary education in the Netherlands.

### 3.1. Context of the Study: Dutch Secondary Education

In the Netherlands, students can follow several secondary education tracks: (1) pre-vocational secondary education (VMBO, 4 years), (2) senior general secondary education (HAVO, 5 years), and pre-university education (VWO, 6 years) after they complete primary education (age 12–13). The enrolment in secondary education is based on two criteria: (1) student performance in the standardised test (in Dutch: Cito-toets) at the end of primary education, and (2) their teacher’s recommendation. School choice is open and parents choose a school for their children. Students’ school pathways in secondary education are highly differentiated in terms of curricula, prestige, duration, final qualifications, and possible academic destination after secondary education (Sykes & Kuyper, 2013).

Generally, Dutch schools offer multiple tracks. In the first year, students can follow a program that does not differentiate yet between two tracks or more. In the second year, students are assigned to a definitive track based on their performance in the first year. Social class and ethnic group are not distributed evenly across the different tracks in secondary education. The lower track over-represents students of parents with lower levels of education, while the higher tracks under-represent students from lower educational backgrounds (Herweijer, 2009). Regardless of this discrepancy, research suggest that ethnic composition is not related to achievement in secondary education, after taking into account socio-economic composition. School composition accounts for little, if any, of the observed differences in achievement across ethnic/social background (Sykes & Kuyper, 2013).

### 3.2. Research Questions

The current study aims to answer the following research questions:

1. Does students’ level of academic engagement differ between school type, school subjects, gender, and parental education background?
2. To what extent do different aspects of perceived teaching behaviours relate to students’ level of academic engagement?
3. Do student gender and parental level of education moderate the relationship between perceived teaching behaviours and academic engagement?

## 4. Materials and Methods

The current study constitutes a quantitative, cross-sectional survey design that includes multilevel modelling, including hierarchical samples of students, teachers/classrooms, and schools. Our theoretical framework hypothesises that the extent to which beginning teachers are able to provide a responsive learning environment affects student engagement indirectly and that this requires teachers to display skills across a wide range of teaching behaviours. In this paper, we are interested in studying the relationship between student engagement in class and the extent to which beginning teachers display teaching behaviours that are preconditional to providing a responsive learning environment. Since beginning teachers start to work in diverse settings and the average level of student engagement in class can be expected to differ (to the extent to which teachers are able to create a responsive learning environment), we opted for a large-scale data analysis using multilevel modelling to account for differences between classes in the overall level of engagement achieved by teachers.

### 4.1. Participants

Data for this study were taken from a large database containing data about students’ perception of teaching behaviours of beginning teachers in the Netherlands (*n* = 11,973; 647 teachers; 198 schools). The data in this database were collected in the context of a study about the effects of induction on the development of beginning teachers’ teaching behaviours. The composition of the group of students in this study is displayed in Table 1. The large data regarding student perceptions of teaching behaviours and self-reported engagement were the appropriate choice to answer the research questions aimed at identifying general patterns across the Dutch secondary education population, in the context of beginning teachers. Statistically, the large data ensured the power for detecting more reliable estimates of the (main) effects of different teaching behaviour dimensions. The sample covered two common education types in the Netherlands across different regions of the country, with teachers adopting various pedagogical practices.

### 4.2. Instruments and Variables

Student perceptions of teaching behaviours during lessons were assessed by means of the My Teacher Questionnaire (MTQ, Maulana & Helms-Lorenz, 2016). The MTQ comprises 41 items that allow students to rate the relative frequency of teaching behaviours in six dimensions: Learning Climate (LM) (5 items, e.g., “My teacher answers my questions,” α = 0.75); Classroom Management (ORG) (8 items, e.g., “My teacher applies clear rules,” α = 0.83); Clarity of Instruction (CLR) (7 items, e.g., “My teacher explains the purpose of the lesson clearly,” α = 0.86); Activating Learning (ACT) (10 items, e.g., “My teacher encourages me to think for myself,” α = 0.86); Differentiated Instruction (DIF) (4 items, e.g., “My teacher knows what I have difficulty with,” α = 0.79); and Teaching Learning Strategy (TLS) (7 items, e.g., “My teacher teaches me to check my solutions,” α = 0.85). Response categories were provided on a 4-point Likert scale, ranging from 1 (never) to 4 (often).

Student engagement was measured using two scales developed by Skinner et al. (2009). The Emotional Engagement scale consists of 5 items (e.g., “In this class I feel good,” α = 0.80). The Behavioural Engagement scale also contains 5 items (e.g., “In this class I pay attention,” α = 0.84). Students could pick one of four answers to indicate to what extent each item applied to their engagement during lessons: 1 (never) to 4 (often).

To correct for possible bias due to differences in students’ tendencies to provide socially desirable judgements, we used a social desirability scale in terms of Impression Management (Crowne & Marlowe, 1960). Five items were adapted to assess the extent to which students were concerned with social approval in the classroom. An example item is “I always listen carefully to teachers,” α = 0.60). Each item of the scale is provided with response categories on a 4-point Likert scale (1 = completely disagree, 4 = completely agree). Students’ background variables were coded as follows: student gender (0 = male, 1 = female), student age group (0 = 11–16 years old and 1 = 16 and older), and teacher subject (0 = natural sciences, 1 = languages and social sciences). Parental ‘level of education’ was coded as a set of dummy variables contrasting different levels of advanced education with primary education level as a reference category. School type was coded as dummy variables with general education as a reference category.

### 4.3. Analysis Strategy

Because the data were naturally and systematically structured in a hierarchical order (i.e., students nested within teachers/classes and teacher/classes nested within schools), multilevel modelling was appropriate for analysing this type of data (Snijders & Bosker, 2012). This modelling improved the accuracy of the fixed effect and standard error estimates and reduced the inflation of lower-level variance estimates (Chen, 2012).

Our analysis strategy involved the comparison of increasingly complex three-level random intercept models in which teaching behaviours and interactions with background variables were included. Our base model included fixed effects of school type, school subject, and background variables on the engagement variables. This model was used to assess gaps in students’ level of engagement between school types, school subjects, male and female students, students of different ages, and parents with different levels of education (Research question 1).

Assuming a difference between schools and classes, it then became relevant to examine in detail which teaching behaviours affect student engagement most saliently (Research question 2), and whether these effects either diminish or increase the gap between different categories of students and contexts (Research question 3). We adjusted the learning environment variables by means of grand mean centring and specified the effects of the variables and interactions as fixed factors. We included students’ scores on the social desirability scale as a covariate to correct for bias in students’ perceptions. An empty model (a model without predictors and covariates) was used to calculate the percentage of variance explained by the more complex models. The modelling was performed using a stepwise procedure and separately for each dimension of teaching behaviours. Significant predictors at *p* < 0.05 were retained. The fixed effects in the model were tested by using t-ratio coefficients for a significant effect of a variable (Snijders & Bosker, 2012).

## 5. Results

### 5.1. Preliminary Analysis: Descriptive Results

Table 2 displays the descriptive statistics of students’ self-reports about their engagement and their perceptions of their teacher’s behaviours during lessons. The mean score for Emotional engagement is 2.92 (*SD* = 0.68) and for Behavioural Engagement is 3.01 (*SD* = 0.55) respectively, indicating that students, on average, stated they mostly felt at ease during lessons and paid attention to teachers’ instructions. Concerning the different dimensions of teaching behaviour, higher means indicated that these were more often evident during lessons for the students. From Table 2, it can be concluded that, generally according to the students, teachers provided stimulating learning climate (*M* = 3.23, *SD* = 0.56) and clear instruction in a safe environment in most lessons (*M* = 3.18, *SD* =0.55) while managing their classroom efficiently (*M* = 3.34, *SD* = 0.50). Concerning the more student-centred teaching behaviour dimensions—Activating Learning (*M* = 2.97, *SD* = 0.58), Differentiated Instruction (*M* = 2.78, *SD* = 0.66), and Teaching Learning Strategies (*M* = 2.54, *SD* = 0.65)—the mean values indicated that about half of the students experienced these teaching behaviours as relatively absent during lessons. The mean on the social desirability scale was 3.01 (*SD* = 3.01), which indicated a high level of social desirability.

### 5.2. Students’ Perceptions of Teaching Behaviours and Student Engagement

The relationships between student’s perceived teaching behaviours in the learning environment and students’ self-reported Emotional and Behavioural Engagement and the effects of students’ characteristics and curriculum factors on student engagement were investigated by means of a series of increasingly complex random intercept models to assess main and interaction effects.

#### 5.2.1. Emotional Engagement

The results of Model 0 (Empty Model) showed that there was a very large difference between student variation in Emotional Engagement (74%) compared to between class (25%) and between school (1%) variation. This meant that differences in Emotional Engagement between students within a class were substantially large. Differences in Emotional Engagement between classes within a school were also noticeably large.

Table 3 displays the results of the two core models. The results of model 1 (Covariate Model) indicated that Emotional Engagement marginally depended on background factors. Student gender explained differences in Emotional Engagement, with male students having higher scores than female students (*p* < 0.01). Vocational education and the school subject category ‘languages and social sciences’ had significantly positive relationships with Emotional Engagement (*p* < 0.01), showing that vocational education students and language and social sciences students reported higher Emotional Engagement than general education and science subject students. The effects of parental education and student age were not significant (*p* > 0.05). In sum, student gender, school type, and school subject appear to be important predictors of Emotional Engagement, but not parental education and student age.

In addition to Model 1, Model 2 (Full Model) also included an assessment of teaching behaviour dimensions. The results of model 2 indicated that five out of six dimensions of teaching behaviour had distinct positive effects on Emotional Engagement (*ps* < 0.05), even after controlling for several background variables and social desirability. The only dimension not related to Emotional Engagement was Clarity of Instruction. Especially Activating Learning and to a lesser extent Classroom Management had considerable positive effects on Emotional Engagement. Additional analysis separating the six dimensions of teaching behaviour and Emotional Engagement, controlling for several background variables and social desirability, revealed that Clarity of Instruction had a significant and positive effect on Emotional Engagement (see Appendix A. Activating Learning appeared to be the primary driver of Emotional Engagement, suggesting that the effect of Clarity of Instruction depended on the presence of Activating Learning.

In sum, all six dimensions of teaching behaviour are important for Emotional Engagement, but Activating Learning is by far the strongest predictor. It is also important to note that the effect of background variables was not significant anymore after the six dimensions of teaching behaviour were included in the model. This means that the six dimensions of teaching behaviour are stronger predictors of, and more directly related to, Emotional Engagement.

Further investigation of potential differential effects by means of a model comprising interactions between background variables and the six dimensions of teaching behaviour revealed no significant interaction effects. This interaction model showed a less good statistical fit of the differences between students concerning Emotional Engagement than Model 2, which indicated that the importance of perceived teaching behaviours on student engagement appeared to be generalisable for all students.

Overall, results suggested that the effect of background variables diminished when the six teaching behaviour dimensions were added. This indicated that differences in Emotional Engagement mainly depended on students’ perceptions of their teachers’ teaching behaviours, and less on their personal and contextual backgrounds.

#### 5.2.2. Behavioural Engagement

The results of Model 0 (Empty Model) showed that there was a substantial difference between student variation in Behavioural Engagement (81%) compared to between class (17%) and between school (2%) variation. This meant that differences in Behavioural Engagement between students within a class were substantially large. Differences in Emotional Engagement between classes within a school were not negligible.

Table 4 displays the results of our analyses. The results of Model 1 indicated that students’ background factors related significantly to Behavioural Engagement. Students with parents with only a primary education background reported significantly less Behavioural Engagement than students with parents from other educational backgrounds (*p* < 0.05). Female students showed less Behavioural Engagement than male students (*p* < 0.01). School type and school subject had significantly positive relationships with Behavioural Engagement (*p* < 0.001), indicating that vocational education students and language and social sciences students reported higher Behavioural Engagement than general education and science subject students. In sum, student gender, school type, school subject, and parental education level appear to be important predictors of Behavioural Engagement, but not student age.

Model 2 included the effects of teaching behaviours. The results of this model indicated that four out of six teachers’ teaching behaviours, Classroom Management, Clarity of Instruction, Teaching Learning Strategies, and especially Activating Learning, had positive links with Behavioural Engagement (*ps* < 0.001), even after controlling for several background variables and social desirability. The effect of Teaching Learning Strategies on Behavioural Engagement was not significant (*p* > 0.05), while that of Learning Climate was negative. A subsequent model evaluating interactions between background variables and the six teaching behaviours showed a less favourable statistical fit than Model 2 and revealed no significant differences in the nature and strength of the relationship between teaching behaviours and Behavioural Engagement, indicating that the positive links between students’ perceptions of teacher behaviours and student engagement were generalisable for all students.

Additional analysis separating the six dimensions of teaching behaviour and Behavioural Engagement revealed that, controlling for several background variables and social desirability, both Learning Climate and Teaching Learning Strategies had significant and positive effects on Behavioural Engagement. Activating Learning appeared to be the primary driver of Behavioural Engagement, influencing the effects of Learning Climate and Teaching Learning Strategies when modelled together.

In sum, all six dimensions of teaching behaviour are important for Behavioural Engagement, but Activating Learning is by far the strongest predictor. The effect of background variables was not significant anymore after the six dimensions of teaching behaviour were included in the model. This means that the six dimensions of teaching behaviour are stronger predictors of, and more directly related to, Behavioural Engagement.

#### 5.2.3. Effect Size of Teaching Behaviour Dimensions

**Figure 2:**
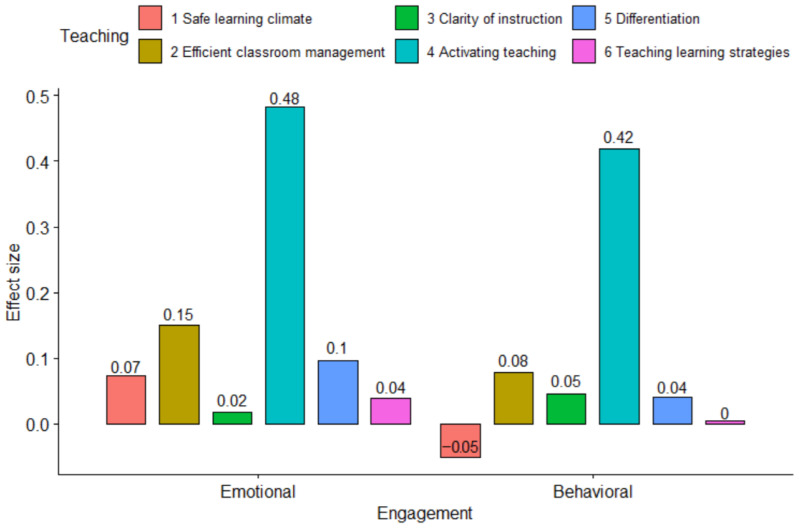
Effect size of different teaching behaviour dimensions.

The effects of the six teaching behaviour dimensions in Model 2 from Table 3 and Table 4 are depicted in Figure 2. Figure 2 shows that the combined effects of the dimensions Classroom Management and Activating Learning amounted to 0.63 on the four-point scales for Emotional Engagement and 0.50 for Behavioural Engagement. These figures indicated a substantial and practically meaningful relationship.

## 6. Conclusions and Discussion

The current study aimed to examine the relationship between student perceptions of teaching behaviours and their academic engagement during lessons in Dutch secondary schools. In so doing, we also examined the differential effectiveness of essential dimensions of teacher behaviours for stimulating specific groups of students to actively engage in learning to achieve mastery of the learning objectives, as equity in the classroom setting is generally considered an important objective of school teaching.

The results of this study showed that students’ level of engagement differed marginally between school type and school subject. Students’ background characteristics had a limited relationship with student engagement (Research question 1). It is nonetheless noteworthy that students with parents who only completed primary education showed consistently somewhat less Behavioural Engagement. This finding contributes to extending the knowledge base regarding the marginal, but not negligible, role of students’ background characteristics in their engagement in learning, from the Dutch beginning teacher context. Past research revealed that higher Behavioural Engagement is associated with a higher likelihood that students will express higher education expectations (van den Broeck et al., 2022; Wang & Eccles, 2012). Higher education expectations are lower among students from low SES families (Tramonte & Willms, 2010). Other research showed that student engagement and achievements are reciprocally related (van den Broeck et al., 2022), which suggests that fostering engagement leads to better achievements, and better achievements dynamically boost engagement. To reduce quality and equity gaps in educational outcomes, it is therefore important to pay extra attention to the (Behavioural) engagement of students from lower SES groups. The question is, how can teachers address this issue in their teaching practices?

The results of this study shed light on how teachers can foster student engagement, particularly in the Dutch secondary education context for beginning teachers. We found that all teaching behaviour dimensions are related to both Emotional Engagement and Behavioural Engagement, albeit at different magnitudes (Research question 2). This finding extends theoretical knowledge by documenting that the six dimensions of teaching behaviour matter for student engagement, but certain dimensions of behaviour matter more than others. Activating Learning appears to be the most salient dimension affecting both Behavioural and Emotional Engagement. Particularly, Activating Learning and Classroom Management together produce substantial effects on both Behavioural and Emotional Engagement. This suggests that in classrooms where students perceive their teachers to provide efficient classroom management more regularly and a more intensive and activating way of teaching and learning, their engagement has, in general, reached a substantial level, for both Emotional and Behavioural Engagement. Our findings support the findings of past studies documenting the importance of active learning for engaging students in learning (O’Neal & Pinder-Grover, 2021; Finelli et al., 2018; Theobald et al., 2020), from the Dutch beginning teacher context. Considering the size of these effects for all students, it is therefore important for school or team leaders to monitor these two dimensions of teaching behaviours and, in the case of poor performance, to discuss options with (beginning) teachers for improvement.

The reasons why Activating Learning strongly predicted student engagement more than other teaching behaviour dimensions remains inconclusive. Nevertheless, there is evidence that active learning is key to promoting student engagement (Hodges, 2020). Certain teacher practices can powerfully induce student engagement. Key active learning practices include designing active learning that serve the learning goals, adopting behaviours that promote trust and reduce student resistance, and describing activities to elicit constructive and interactive engagement. Active learning can empower students to take their learning to a whole new level (Hodges, 2020).

Although most teachers succeed in activating the learning of their students, about half of the students indicated that their teachers did not teach them learning strategies. This probably meant that most students had to discover these by themselves, which in turn meant that they might face difficulties in engaging and regulating their own learning. It is therefore important to include in teacher training more exercises to assess student learning strategies, to discuss improvements, and suggest more effective learning strategies when needed (cf. Voskamp et al., 2022).

The effect of Learning Climate, which is interconnected with that of Activating Learning, remains paramount. Past research showed that students’ perceiving a classroom climate focused on the learning process, which puts more emphasis on interaction, is beneficial for student engagement, while a classroom climate that focuses on learning results is detrimental to student engagement (Gutierrez et al., 2019). Classroom climates that foster student engagement are characterised by caring, collaborative, and inclusive practices (Sanchez-Hernandez et al., 2018; Song & Kim, 2016). Higher levels of relatedness support are related to higher levels of engagement (Cents-Boonstra et al., 2021). Asking motivating questions and providing positive feedback and support during learning are associated with positive engagement (Cents-Boonstra et al., 2022). Greater student engagement is garnered when teachers enhance the quality of interactions and promote interactive dialogic relationships with students (Lim et al., 2023). Teachers’ provision of a safe and stimulating learning climate provides a basis for learning to occur, which seems to serve as a pre-condition for teachers to exhibit more advanced teaching skills, such as Differentiation and Teaching Learning Strategies (Maulana et al., 2015; van de Grift et al., 2014; van der Lans et al., 2016).

Although Differentiation had a unique effect on student engagement, its magnitude was not substantial. The same also applied to Teaching Learning Strategies, which depends on Activating Learning. This might be due to the fact that teachers did not engage in these teaching behaviours as regularly and frequently during their lessons as the other teaching behaviour dimensions. Past research showed that, particularly in Dutch secondary education, and in many parts of the world generally, teachers exhibited low levels of Differentiated Instruction and Teaching Learning Strategies (Maulana et al., 2021; Maulana et al., 2023b). Certain (high) levels of these two advanced dimensions of teaching behaviour are needed in order to reveal their substantial influence on student outcomes (e.g., engagement). The literature demonstrates that both Differentiation and Teaching Learning Strategies are important predictors of student achievement (Bybee et al., 2006; Hattie, 2008; OECD, 2019). A meta-analysis study further showed that Differentiation has a relatively substantial effect on student achievement in secondary education (Smale-Jacobse et al., 2019).

Finally, we did not uncover a differential effectiveness of teachers’ teaching behaviours on student engagement in Dutch secondary schools for students with different background features (Research question 3). This means that all relevant dimensions of teaching behaviour are equally important to increase student engagement for students of all backgrounds, in terms of school type, school subject, student gender, student age, and parental education level. This finding suggests that the importance of the six teaching behaviours for student engagement seems to be universal for students with various background characteristics, particularly for the Dutch secondary education context.

One many argue that the non-significant interaction effects found in this study may be partly due to measurement constraints or restricted variance. We used scale scores in a large sample of schools and students, so restricted variance due to measurement constraints can be ruled out. The incorporation of six teaching dimensions that are highly correlated could have induced variance restriction, which in turn could have potentially hidden interaction effects to a certain extent. However, a large part of the variance (over 50%) could be explained, so restricted variance does not seem to be an issue in this study.

**Figure 3:**
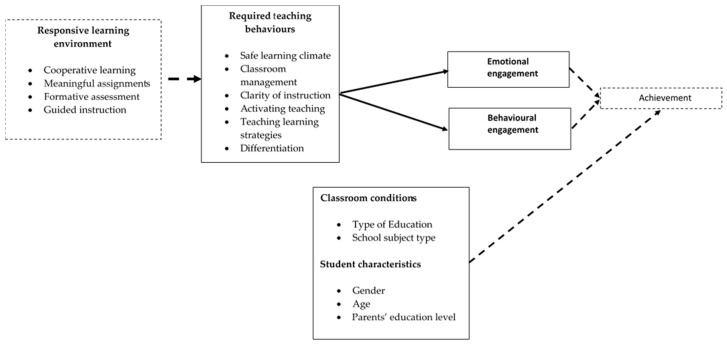
Empirical model depicting the relationships between teaching behaviours, student engagement, learning outcomes, and background variables. (Solid line = tested variables, dashed line = not tested variables).

To conclude, the findings of the current study partly confirmed our conceptual model (see Figure 1), providing evidence that the six dimensions of teaching behaviour are strongly more important than student and contextual backgrounds for explaining differences in Emotional and Behavioural Engagement, and that student and contextual backgrounds do not play a role in moderating the link between teaching behaviours and student engagement (see Figure 3). Our findings confirm past studies showing the general importance of high-quality learning environments for academic engagement from the student lens (Cinches et al., 2017). Intensive Activating Learning and Classroom Management appear to be two most salient predictors of both engagement types, suggesting that contemporary learning environments should promote more active teaching and learning processes coupled with efficient classroom management, without ignoring other components of learning environment support, particularly Differentiation and Teaching Learning Strategies.

### 6.1. Limitations of the Study

This study is subject to several limitations. Although the study employed large data from one particular highly-recognized education system, the nature of the study is correlational and cross-sectional. Hence, the results of the study should be limited and interpreted as correlational, depicting results from single measurement only. Although the term “effect” was used to refer to the nature of the statistical analysis employed (e.g., multilevel modelling), this should be interpreted in terms of relationships between the variables included. Additionally, the sample of the current study is predominantly beginning teachers. As we expect variations in the skill level development of teaching behaviours as a function of teaching experience, it is recommended that future studies address more diverse and representative teacher populations in terms of teaching experience.

Moreover, our study focuses on the impact of teacher behaviours without examining specific details about the actual learning environment that provides the setting in which teachers apply these behaviours. Although in the Netherlands the teaching settings are rather similar due to the fact that most teachers use lesson packages that stipulate comparable ways of presenting instruction in school subject content throughout the school year and our multilevel analysis procedures statistically corrected for differences in these settings, our results do not provide insight into how alternative variations in learning environment settings may result in more or less activating learning and increased student engagement for specific school subjects. Further research linking alternative settings to teachers‘ behaviours during lessons are therefore necessary to provide these insights. Barlow and Brown (2020), for instance, indicated that stimulating interactivity with peers, what they considered to be the deepest mode of cognitive engagement, was related to instructional practices that encourage deeper learning of course material. We therefore conclude that there is a need for ongoing research to study the interplay of teachers’ instructional practices and student engagement.

Another limitation stems from the fact that our study focuses on two aspects of student engagement, while several other studies focused on three aspects, also including cognitive engagement (e.g., Fredricks et al., 2004). Future studies can benefit from insights into how the six dimensions of teaching behaviour relate to cognitive engagement, and to what extent the effects differ compared to the two engagement dimensions studied herein. Due to a lack of data about students’ learning approaches and achievements, we could not assess the relationship between teacher behaviours and student achievements, nor moderation by student engagement. This avenue is recommended for future research. Finally, the current study relied mainly on self-reported data. Future research should aim to include classroom observational data to understand the complex interplay between classroom practices and student engagement more comprehensively.

### 6.2. Implications of the Study and Future Directions

The current study has several implications for educational practice, teacher education, policy, and future directions. The findings that effective Classroom Management and Activating Learning both have substantial effects on student engagement during lessons in class means that induction arrangements for beginning teachers in schools, next to providing emotional support, should focus on observing their performance in these teaching behaviour dimensions and on providing support when teachers show shortcomings in these two dimensions. For teacher education, these three teaching behaviour dimensions should continue to be core topics in preparing future teachers, so that graduates will reach good mastery of these pedagogic-didactic skills. As the effect size of Classroom Management and Activating Learning for student engagement altogether was considerable, these teaching behaviour dimensions should be considered in teacher professional development policies as part of the starting quality of (Dutch) secondary school teachers.

The fact that Differentiated Instruction and Teaching Learning Strategies received relatively low scores indicates that many teachers need further professionalisation in these areas. These teacher behaviours are elemental in transferring control over learning from the teacher to the student and in providing opportunities for developing metacognitive skills that allow students to become autonomous learners. For teacher education, it is important these two dimensions of teaching behaviour are incorporated to some extent in the education program so that graduates are at least aware of the importance of these higher-level teaching skills. Teacher professional development policies can then be directed toward an expert teacher level, characterised by mastering the six dimensions of teaching behaviour across their professional practice.

How to improve these teaching behaviours can be approached from different angles. When teachers show shortcomings in only one behavioural dimension, training in this dimension may be the most appropriate option. When different dimensions are concerned, focusing on adopting a different instruction model may turn out to be a more effective approach. The fact that many students perceive little evidence of teaching behaviours that support their own agency in learning to achieve mastery of the learning objectives might be a persuasive argument for introducing a new education concept or practice, such as formative assessment or cooperative learning, with possible advantages of creating learning environments in their schools that diminish inequity among students. The problem that school leaders should then solve is how to promote effective guided instruction and formative assessment practices in their schools and to aid beginning teachers to become more proficient in creating a mastery-oriented learning environment that removes inequity among students during their school careers. Differentiation, which is shown to be the most complex teaching skill to master by teachers (Maulana et al., 2023b), can address this inequity issue. Targeted interventions to improve differentiation practices should be part of teacher continuous professional development after the first phase of induction trajectories.

Furthermore, Gencoglu et al. (2025a, 2025b) indicated variation in the relationship between the six dimensions of teaching behaviour and student engagement across education systems. This means that although the literature highlights the importance of the six dimensions of teaching behaviour for student outcomes, the main drivers of student engagement seem to differ depending on the education system (Maulana et al., 2023c). This suggests that the contribution of certain teaching behaviours to learning seems to relate to the values and culture of which the teaching practice is part (Gencoglu et al., 2025a, 2025b). It is therefore beneficial to replicate this study in other contexts and education systems to establish a knowledge base about what is effective and which teaching dimensions are more salient in determining student engagement, learning, and outcomes across educational contexts.

Finally, we did not find significant interaction effects between background variables included in the link between perceived teaching behaviours and engagement, suggesting that the powerful effects of Classroom Management and Activating Learning seem to be shared across student backgrounds in Dutch secondary education. Nevertheless, the inclusion of background factors in the current study was rather limited. In addition, the way we measured the constructs (e.g., self-reports) may contribute to the findings. Future studies should consider including other background factors, such as prior achievement, socio-economic status, and school/class composition, and other more objective methods, such as classroom observations, in examining the link between teaching behaviours and student engagement.

## Figures and Tables

**Table 1:** Descriptive statistics of the sample composition.

Variable	Category	Percentage (%)
School Type		
	General Education	92
	Vocational Education	8
School Subject		
	Science subjects	32
	Language and social science subjects	68
Gender		
	Male students	48
	Female Students	52
Parental Education Level		
	Primary school	8
	Lower high school	27
	Upper high school	13
	Advanced vocational	24
	University education	28
Age Category		
	≤16 years old	92
	>16 years old	8

**Table 2:** Descriptive statistics of the scales used in this study; means and standard deviations.

Scale	Mean	Standard Deviation
Teaching Behavior Dimension		
Learning Climate	3.23	0.56
Classroom Management	3.34	0.50
Clarity of Instruction	3.18	0.55
Activating Learning	2.97	0.58
Differentiation	2.78	0.66
Teaching Learning Strategies	2.54	0.65
**Student Engagement Dimension**		
Emotional Engagement	2.92	0.68
Behavioral Engagement	3.01	0.55
**Social Desirability**	3.01	0.41

Note. Bold letters represent main constructs.

**Table 3:** Results from the multilevel analyses for Emotional Engagement.

	Empty Model		Model 1 (Covariate)		Model 2 (Full)	
	Estimate	S.E.	Estimate	S.E.	Estimate	S.E.
(Intercept)	2.925 ***	0.016	2.867 ***	0.026	2.921 ***	0.019
School Type ^1^			0.108 **	0.040	0.003	0.021
School Subject ^2^			0.091 **	0.029	0.010	0.014
Gender ^3^			−0.032 **	0.010	−0.006	0.008
**Parental Education Level** ^4^						
Lower High School			0.021	0.022	−0.023	0.017
Upper High School			0.022	0.024	−0.009	0.018
Advanced Vocational			0.004	0.022	−0.034 *	0.017
University Education			0.020	0.022	−0.006	0.017
Age Category ^5^			0.003	0.024	0.010	0.017
Social Desirability			0.553 ***	0.013	0.254 ***	0.010
Learning Climate					0.073 ***	0.013
Classroom Management					0.151 ***	0.017
Clarity of Instruction					0.017	0.015
Activating Learning					0.483 ***	0.016
Differentiation					0.096 ***	0.011
Teaching Learning Strategies					0.039 ***	0.010
**Variance Components**						
School Level	0.0061		0.0019		2.5 × 10^−5^	
Teacher Level	0.1164		0.0938		0.0150	
Residual	0.3401		0.2937		0.1796	
Percentage of Variance Explained	-		13.7		47.2	
Loglikelihood	−1163.9		−10,282.8		−7050.1	

Reference categories: ^1^ General education, ^2^ Science subjects, ^3^ Male students, ^4^ Primary education level, ^5^ 16 years or less; Significance levels: *** *p* < 0.001, ** *p* < 0.01, * *p* < 0.05.

**Table 4:** Results from the multilevel analyses for Behavioural Engagement.

	Empty Model		Model 1 (Covariate)		Model 2 (Full)	
	Estimate	S.E.	Estimate	S.E.	Estimate	S.E.
(Intercept)	3.017 ***	0.012	2.920 ***	0.024	2.971 ***	0.018
School Type ^1^			0.098 ***	0.029	0.024	0.019
School Subject ^2^			0.066 ***	0.020	0.012	0.012
Gender ^3^			−0.027 **	0.009	−0.009	0.008
**Parental Education Level** ^4^						
Lower High School			0.071 ***	0.018	0.045 **	0.016
Upper High School			0.062 **	0.020	0.048 **	0.017
Advanced Vocational			0.039 *	0.018	0.016	0.016
University Education			0.058 **	0.019	0.041 *	0.016
Age Category ^5^			−0.020	0.020	−0.046 **	0.016
Social Desirability			0.525 ***	0.011	0.332 ***	0.010
Learning Climate					−0.051 ***	0.012
Classroom Management					0.079 ***	0.016
Clarity of Instruction					0.046 ***	0.014
Activating Learning					0.418 ***	0.015
Differentiation					0.040 ***	0.010
Teaching Learning Strategies					0.004	0.010
**Variance Components**						
School Level	0.0061		0.0032		0.0022	
Teacher Level	0.0523		0.0386		0.0086	
Residual	0.2512		0.209		0.1592	
Percentage of Variance Explained	-		19.0		45.1	
Loglikelihood	−9233.6		−8122.3		−6283.9	

Reference categories: ^1^ General education, ^2^ Science subjects, ^3^ Male students, ^4^ Primary education level, ^5^ 16 years or less; Significance levels: *** *p* < 0.001, ** *p* < 0.01, * *p* < 0.05.

## Data Availability

Data are unavailable due to privacy or ethical restrictions.

## Appendix A

### Supplementary Analysis

**Table A1:** Results from the multilevel analyses with separate teaching behaviour dimensions and Emotional Engagement.

	Model 0	Model 1	Model 2a	Model 2b	Model 2c	Model 2d	Model 2e	Model 2f	Model 3
Predictors	Estimates	Estimates	Estimates	Estimates	Estimates	Estimates	Estimates	Estimates	Estimates
(Intercept)	2.92 ***	2.87 ***	2.89 ***	2.91 ***	2.89 ***	2.89 ***	2.89 ***	2.87 ***	2.90 ***
School Type: Vocational education		0.11 **	0.06 *	0.06 *	0.05	0.02	0.03	−0.02	0.01
School Subject Type: Non-science		0.09 **	0.03	0.02	0.02	0.02	0.04	0.05 *	0.01
Gender: Girls		−0.04 ***	−0.03 ***	−0.03 ***	−0.01	−0.01	−0.00	0.00	−0.01
Educational Level Parents: Higher Education		−0.00	0.00	−0.00	0.01	0.00	−0.00	0.01	0.00
Age Group: >16 years		0.00	0.03	0.04	0.03	0.01	−0.01	−0.01	0.01
Social Desirability		0.56 ***	0.35 ***	0.34 ***	0.36 ***	0.28 ***	0.37 ***	0.40 ***	0.26 ***
Learning Climate			0.61 ***						0.08 ***
Classroom Management				0.74 ***					0.15 ***
Clear Instruction					0.65 ***				0.02
Activating Learning						0.76 ***			0.48 ***
Differentiation							0.51 ***		0.09 ***
Teaching Learning Strategies								0.49 ***	0.04 ***
**Random Effects**									
σ^2^	0.34	0.29	0.23	0.22	0.23	0.19	0.22	0.24	0.18
τ_00_	0.12 _TeacherID:SchoolID_	0.09 _TeacherID:SchoolID_	0.03 _TeacherID:SchoolID_	0.03 _TeacherID:SchoolID_	0.03 _TeacherID:SchoolID_	0.02 _TeacherID:SchoolID_	0.03 _TeacherID:SchoolID_	0.04 _TeacherID:SchoolID_	0.01 _TeacherID:SchoolID_
	0.01 _SchoolID_	0.00 _SchoolID_	0.00 _SchoolID_	0.00 _SchoolID_	0.00 _SchoolID_	0.00 _SchoolID_	0.00 _SchoolID_	0.00 _SchoolID_	0.00 _SchoolID_
ICC	0.26	0.24	0.13	0.13	0.12		0.13	0.15	
N	647 _TeacherID_	647 _TeacherID_	647 _TeacherID_	647 _TeacherID_	647 _TeacherID_	647 _TeacherID_	647 _TeacherID_	647 _TeacherID_	647 _TeacherID_
	198 _SchoolID_	198 _SchoolID_	198 _SchoolID_	198 _SchoolID_	198 _SchoolID_	198 _SchoolID_	198 _SchoolID_	198 _SchoolID_	198 _SchoolID_
Observations	11,634	11,634	11,634	11,634	11,634	11,634	11,634	11,634	11,634
Marginal R^2^/Conditional R^2^	0.000/0.263	0.124/0.338	0.397/0.474	0.433/0.504	0.412/0.483	0.568/NA	0.395/0.475	0.357/0.452	0.587/NA
Deviance	21,760.756	19,976.954	16,497.850	16,055.241	16,426.473	14,016.855	16,434.147	17,032.894	13,591.383
AIC	21,775.158	20,040.338	16,574.447	16,131.800	16,503.143	14,096.352	16,511.124	17,109.161	13,716.018
log-Likelihood	−10,883.579	−10,010.169	−8276.224	−8054.900	−8240.572	−7037.176	−8244.562	−8543.581	−6842.009

Note: * *p* < 0.05, ** *p* < 0.01, *** *p* < 0.001, NA = not applicable.

**Table A2:** Results from the multilevel analyses with separate teaching behaviour dimensions and Behavioural Engagement.

	Model 0	Model 1	Model 2a	Model 2b	Model 2c	Model 2d	Model 2e	Model 2f	Model 3
Predictors	Estimates	Estimates	Estimates	Estimates	Estimates	Estimates	Estimates	Estimates	Estimates
(Intercept)	3.02 ***	2.99 ***	3.01 ***	3.02 ***	3.01 ***	3.01 ***	3.00 ***	2.99 ***	3.01 ***
School type: Vocational education		0.09 **	0.06 *	0.06 *	0.05 *	0.02	0.04	0.00	0.02
School Subject Type: Non-science		0.06 **	0.03	0.02	0.02	0.02	0.03	0.04 *	0.01
Gender: Girls		−0.03 **	−0.03 ***	−0.03 ***	−0.01	−0.01	−0.01	−0.01	−0.01
Education Level Parents: Higher Education		−0.01	−0.01	−0.01	−0.01	−0.01	−0.01	−0.01	−0.01
Age Group: >16 years		−0.03	−0.03	−0.02	−0.03	−0.05 **	−0.05 **	−0.05 **	−0.05 **
Social Desirability		0.53 ***	0.40 ***	0.38 ***	0.39 ***	0.34 ***	0.41 ***	0.43 ***	0.33 ***
Learning Climate			0.36 ***						−0.05 ***
Classroom Management				0.47 ***					0.08 ***
Clear Instruction					0.43 ***				0.05 **
Activating Learning						0.50 ***			0.42 ***
Differentiation							0.32 ***		0.04 ***
Teaching Learning Strategies								0.31 ***	0.00
**Random Effects**									
σ^2^	0.25	0.21	0.19	0.18	0.18	0.16	0.18	0.19	0.16
τ_00_	0.05 _TeacherID:SchoolID_	0.04 _TeacherID:SchoolID_	0.02 _TeacherID:SchoolID_	0.01 _TeacherID:SchoolID_	0.01 _TeacherID:SchoolID_	0.01 _TeacherID:SchoolID_	0.02 _TeacherID:SchoolID_	0.02 _TeacherID:SchoolID_	0.01 _TeacherID:SchoolID_
	0.01 _SchoolID_	0.00 _SchoolID_	0.00 _SchoolID_	0.00 _SchoolID_	0.00 _SchoolID_	0.00 _SchoolID_	0.00 _SchoolID_	0.00 _SchoolID_	0.00 _SchoolID_
ICC	0.19	0.17	0.11	0.09	0.08	0.07	0.10	0.10	0.07
N	647 _TeacherID_	647 _TeacherID_	647 _TeacherID_	647 _TeacherID_	647 _TeacherID_	647 _TeacherID_	647 _TeacherID_	647 _TeacherID_	647 _TeacherID_
	198 _SchoolID_	198 _SchoolID_	198 _SchoolID_	198 _SchoolID_	198 _SchoolID_	198 _SchoolID_	198 _SchoolID_	198 _SchoolID_	198 _SchoolID_
Observations	11,634	11,634	11,634	11,634	11,634	11,634	11,634	11,634	11,634
Marginal R^2^/Conditional R^2^	0.000/0.188	0.162/0.302	0.301/0.376	0.349/0.407	0.349/0.402	0.437/0.474	0.319/0.388	0.300/0.371	0.443/0.479
Deviance	17,997.844	15,785.839	14,162.866	13,598.009	13,614.639	12,245.577	13,878.118	14,170.458	12,155.865
AIC	18,012.787	15,852.614	14,241.236	13,676.838	13,693.869	12,326.214	13,957.202	14,249.395	12,282.034
log-Likelihood	−9002.394	−7916.307	−7109.618	−6827.419	−6835.934	−6152.107	−6967.601	−7113.698	−6125.017

Note: * *p* < 0.05 ** *p* < 0.01 *** *p* < 0.001.
